# Reovirus µ2 residue 208 drives SRSF2-dependent nuclear hyperaccumulation of IRF9

**DOI:** 10.17912/micropub.biology.002201

**Published:** 2026-07-27

**Authors:** Efraín E. Rivera-Serrano

**Affiliations:** 1 Department of Biology, Elon University, Elon, NC, United States

## Abstract

Mammalian reovirus µ2 antagonizes type I interferon (IFN) signaling and is associated with nuclear hyperaccumulation of IRF9. We tested whether µ2 residue 208, which determines strain-specific IFN-β repression and modulation of host cell mRNA splicing, also controls IRF9 relocalization and whether the splicing factor SRSF2 is required. Recombinant and mutant viruses showed that T1L-like Pro208 is required and sufficient for IRF9 nuclear accumulation in L929 cells. In addition, SRSF2 depletion reduced IFN-mediated induction of *Irf7* and *Stat1* , but not *Isg56/Ifit1* , and abolished T3D-S208P-induced IRF9 nuclear accumulation. Thus, reovirus-induced IRF9 relocalization requires both µ2 residue 208 and host SRSF2.

**Figure 1. Reovirus µ2 residue 208 drives nuclear hyperaccumulation of IRF9 in an SRSF2-dependent manner:** (A) L929 cells were infected with the indicated reoviruses at an MOI of 50 PFU per cell for 24 h. Cells were fixed and subjected to immunostaining using antibodies against IRF9 and reovirus mouse antisera (Rivera-Serrano and Sherry, 2017). Nuclei were counterstained with DAPI. Scale bar = 10 µm. Histograms display measured fluorescence intensity for IRF9 (green) and DAPI (blue) along the drawn line in the IRF9 panels using the image processing package Fiji as previously described (Rivera-Serrano et al., 2017a). IRF9 localization was scored in mock cells and in reovirus antigen-positive infected cells from three independent experiments. The number of cells scored per condition was: Mock, n = 196; T1L, n = 203; T3D, n = 216; T3D T1L-M1, n = 244; T1L T3D-M1, n = 211; T3D-S208P, n = 207; and T3D T1L-M1 P208S, n = 224. Data are shown as the percentage of cells with nuclear IRF9 accumulation, averaged across three independent experiments ± standard deviation. Statistical significance was determined using one-way ANOVA with Tukey’s multiple-comparisons test; *P* < 0.05 was considered significant. (B) L929 cells were transfected with either a control non-targeting (NT) siRNA or an SRSF2-specific siRNA for 72 h as described in (Rivera-Serrano et al., 2017b) and stimulated with 1000 U/ml IFN-β for 5 h prior to mRNA harvest for qRT-PCR. Fold induction (average ± standard deviation) is expressed as IFN-treated cells over mock-treated cells for the corresponding siRNA used (*, *P* < 0.01 using a student t-test). Efficiency of SRSF2 knockdown was assessed by immunoblotting. (C) Purity of cytoplasmic and nuclear protein fractions from L929 cells using the NE-PER ^TM^ kit (Thermo Fisher Scientific, #78833) was determined by immunoblotting against α-tubulin (Millipore-Sigma #T6199; 1:10,000) and the transcription factor Sp1 (Santa Cruz Biotech #sc-14027; 1:500). (D) L929 cells were transfected with the indicated siRNA for 72 h, and infected with the indicated reovirus at an MOI of 50 PFU per cell for 24 h. Cytoplasmic and nuclear protein fractions (20 µg/sample) were resolved by SDS-PAGE and subjected to immunoblotting using antibodies against µ2 (1:1,000; (Rivera-Serrano et al., 2017b)), IRF9 (Santa Cruz Biotech #sc-10793; 1:100), or β-actin (Santa Cruz Biotech #sc-1615-hrp; 1:3,000). Densitometry values for normalized IRF9 band intensities are expressed relative to the band intensity of “control siRNA, mock infected” for each panel. Statistical significance was determined using Student’s *t* -test for planned comparisons between NT siRNA and SRSF2 siRNA for each virus condition.

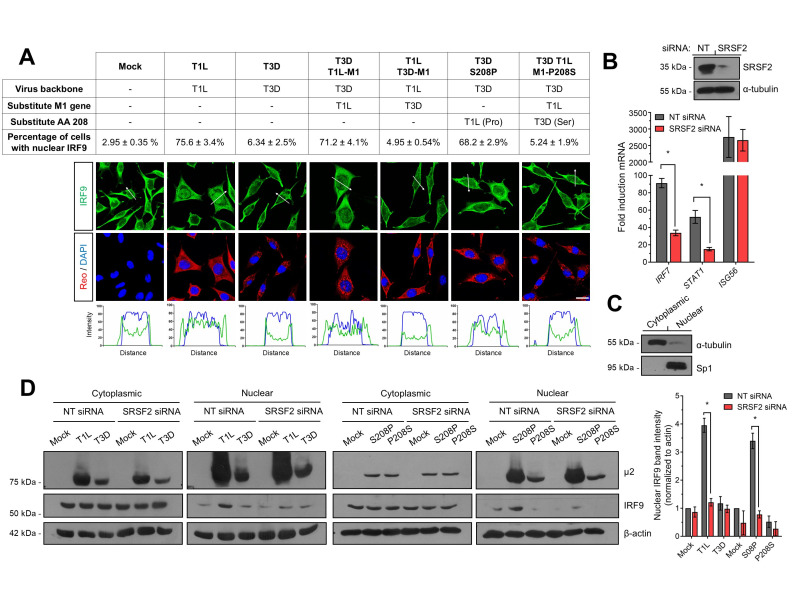



## Description

Type I interferon (IFN-α/β) signaling is a central antiviral defense that induces interferon-stimulated genes (ISGs) through the JAK–STAT pathway. IFN receptor engagement promotes STAT1 and STAT2 phosphorylation, assembly of the ISGF3 complex with IRF9, nuclear translocation of ISGF3, and transcriptional activation of ISGs with antiviral activity (Dalskov et al., 2023, Schneider et al., 2014). Many viruses disrupt this pathway, including mammalian orthoreoviruses (“reoviruses”), nonenveloped double-stranded RNA viruses whose strain-specific host responses have informed studies of viral pathogenesis and oncolysis (García-Sastre, 2017, Müller et al., 2020, Zhu et al., 2023).

The reovirus µ2 protein, encoded by the *M1* gene segment, is a multifunctional viral protein that contributes to strain-specific differences in IFN antagonism. Previous work showed that Type 1 Lang (T1L) represses IFN-stimulated ISG induction, whereas Type 3 Dearing (T3D) does not, and that this phenotype is associated with an unusual nuclear hyperaccumulation of IRF9 (Irvin et al., 2012, Zurney et al., 2009). The T1L-like µ2 residue 208 also determines repression of IFN signaling and modulates viral replication, cytopathic effect, and myocarditis in related systems (Irvin et al., 2012, Sherry et al., 1998). However, whether µ2 residue 208 is required and sufficient for IRF9 nuclear hyperaccumulation, and whether this phenotype depends on host factors that regulate µ2 localization, remained unclear.

We first infected L929 cells with recombinant T1L and T3D viruses, *M1* single-gene reassortant viruses, and single-amino-acid mutants that alter µ2 residue 208. IRF9 was predominantly cytoplasmic in mock-infected cells and in cells infected with T3D, but accumulated in the nucleus in a majority of T1L-infected cells, as determined by scoring IRF9 localization in reovirus antigen-positive cells from three independent experiments ( Fig. 1A ). Quantification of 60–90 cells per condition, per independent experiment showed that approximately 75.6% of T1L-infected cells exhibited nuclear IRF9 accumulation, whereas this phenotype was observed at low frequency in mock- or T3D-infected cells. Additional representative images of T1L-infected L929 cells and no-primary-antibody controls are provided in Extended Data 1A-B. Consistent with the phenotype observed in L929 cells, T1L infection also promoted nuclear accumulation of IRF9 in human AD-293 cells (Extended Data 1C), supporting that this effect is not limited to a single cell type. Consistent with prior mapping of this phenotype to the *M1* segment, T3D carrying the T1L *M1* gene induced IRF9 nuclear accumulation, whereas T1L carrying the T3D M1 gene did not. Substitution of T3D µ2 residue 208 with the T1L-like residue (T3D-S208P) was sufficient to induce IRF9 nuclear accumulation, whereas reverting residue 208 in T3D T1L-M1 to the T3D-like residue (T3D T1L-M1 P208S) eliminated this phenotype. These data indicate that a single µ2 polymorphism determines strain-specific IRF9 relocalization during infection.

Because µ2 interacts with or alters nuclear RNA-processing factors, and because SRSF2 is required for µ2 nuclear localization (Boudreault et al., 2016, Boudreault et al., 2022, Rivera-Serrano et al., 2017b), we next asked whether SRSF2 depletion altered IFN-mediated transcriptional responses. We transfected L929 cells with control or SRSF2-specific siRNA and measured IFN-β-mediated induction of selected IFN-responsive transcripts ( Fig. 1B ). Specifically, we quantified *Irf7* and *Stat1* because these IFN-responsive transcripts were previously shown to be strongly repressed by T1L during IFN-β stimulation (Zurney et al., 2009) and therefore provide continuity with the phenotype examined in the prior study. We also measured *Isg56/Ifit1* , which was not repressed by T1L in that study (Zurney et al., 2009), possibly because *Isg56/Ifit1* can also be induced directly by IRF3 (Grandvaux et al., 2002). SRSF2 knockdown reduced IFN-mediated induction of *Irf7* and *Stat1* by approximately 2.5-fold and 3.0-fold, respectively, but did not similarly reduce induction of *Isg56/Ifit1* ( Fig. 1B ). These data suggest that SRSF2 depletion affects a subset of IFN-responsive transcripts measured here, while also preventing a clean direct test of whether SRSF2 specifically disrupts reovirus-mediated repression of IFN signaling.

We therefore used IRF9 nuclear hyperaccumulation as a marker of reovirus-mediated IFN antagonism. SRSF2 depletion did not detectably alter IRF9 localization in mock-infected cells and did not reduce cytoplasmic IRF9 levels. In control siRNA-treated cells, T1L induced nuclear IRF9 accumulation, whereas T3D did not ( Fig. 1D ). In contrast, T1L-induced IRF9 nuclear hyperaccumulation was abolished in SRSF2-depleted cells. The same SRSF2 dependence was observed for T3D-S208P, whereas the non-accumulating mutant T3D T1L-M1 P208S did not induce nuclear IRF9 accumulation under either siRNA condition. These data show that SRSF2 is required for µ2 residue 208-dependent IRF9 relocalization during reovirus infection.

Together, these findings define a functional link between the reovirus µ2 polymorphism at residue 208, SRSF2-dependent control of µ2 nuclear behavior, and nuclear hyperaccumulation of IRF9. The results support a model in which reovirus-induced IRF9 relocalization requires a host nuclear environment regulated by SRSF2, rather than simply increased µ2 abundance or stability. A limitation is that these experiments were performed in L929 cells using siRNA-mediated depletion, and SRSF2 depletion itself reduced IFN-stimulated ISG induction. Thus, the data establish a requirement for SRSF2 in IRF9 nuclear hyperaccumulation but do not distinguish whether SRSF2 acts by controlling µ2 nuclear localization, IRF9 nuclear transport or retention, ISGF3-associated transcriptional events, or broader RNA-processing functions that influence IFN signaling. Future experiments defining how µ2–SRSF2 interactions alter IRF9 dynamics should clarify this unconventional mechanism of reovirus IFN antagonism.

## Methods

**Cells, viruses, and infections** . L929 and AD-293 cells were maintained at 37°C in 5% CO _2_ as previously described (Rivera-Serrano et al., 2017b). Recombinant reoviruses T1L, T3D, T3D T1L-M1, T1L T3D-M1, T3D-S208P, and T3D T1L-M1 P208S were generated and characterized previously (Rivera-Serrano et al., 2017a, Irvin et al., 2012). Virus stocks were titrated by plaque assay on L929 cells. For infections, L929 cells were adsorbed with the indicated viruses at 50 PFU/cell and incubated for 24 h unless otherwise indicated.

**Immunostaining and image analysis** . L929 cells grown on 8-well chamber slides were mock infected or infected with the indicated viruses at 50 PFU/cell for 24 h. Cells were fixed in 2% paraformaldehyde (Electron Microscopy Sciences) in phosphate-buffered saline and permeabilized with 0.25% Triton X-100 (Sigma). Slides were blocked with 5% normal goat serum and subjected to immunostaining using antibodies against IRF9 for immunofluorescence detection (Abcam #ab51639; 1:500) and reovirus mouse antisera (1:5,000) for 1 h at room temperature. No-primary-antibody controls were processed in parallel using the same secondary antibodies and showed minimal background fluorescence (Extended Data 1A). Nuclei were counterstained with DAPI, and slides were preserved with ProLong Gold. Fluorescence intensity plots for IRF9 and DAPI along the indicated line were generated using Fiji. Approximately 200 cells per condition from three independent experiments were scored for IRF9 signal overlapping with DAPI. For Extended Data 1C, AD-293 cells were mock infected or infected with T1L at an MOI of 25 PFU/cell for 24 h and processed for IRF9 and reovirus immunostaining as described above.

**siRNA depletion and IFN-β stimulation** . L929 cells were transfected with 60 nM of a non-targeting control siRNA or SRSF2-specific siRNA (Horizon Discovery, catalog no. D-001810-10 and L-044306, respectively) for 72 h using the transfection reagent RNAiMAX (Thermo Fisher Scientific), as described previously (Rivera-Serrano et al., 2017b). For ISG induction assays, cells were treated with 1000 U/ml recombinant IFN-β for 5 h, and total RNA was harvested using a QIAGEN RNeasy Kit. RNA was treated with RNase-free DNase I, converted to cDNA by reverse transcription, and used for real-time PCR on a LightCycler 480 fluorescence thermocycler. The reaction mixtures contained 1× QuantiTect SYBR green master mix (Qiagen) and 0.3 μM (each) forward and reverse primers as previously described (Zurney et al., 2009). Values were normalized to GAPDH and expressed as fold induction in IFN-β-treated cells relative to mock-treated cells for each siRNA condition.

**Subcellular fractionation and immunoblotting** . For fractionation experiments, L929 cells were transfected with the indicated siRNAs for 72 h and then mock infected or infected with the indicated reovirus at 50 PFU/cell for 24 h. Cytoplasmic and nuclear fractions were prepared using NE-PER Nuclear and Cytoplasmic Extraction Reagents (Thermo Fisher Scientific, #78833). Fraction purity was assessed by immunoblotting for α-tubulin (Millipore-Sigma #T6199; 1:10,000) and Sp1 (Santa Cruz Biotechnology #sc-14027; 1:500). Protein samples (20 µg) were resolved by SDS-PAGE and transferred to a nitrocellulose membrane. Membranes were probed with antibodies against µ2 (1:1,000; (Rivera-Serrano et al., 2017b)), IRF9 (Santa Cruz Biotechnology #sc-10793; 1:100), β-actin (Santa Cruz Biotechnology #sc-1615-hrp; 1:3,000), or SRSF2 (Millipore, #04-1550; 1:1,000). Densitometry was performed and normalized IRF9 band intensities were expressed relative to control siRNA, mock-infected cells within each panel.

**Statistics** . qRT-PCR, immunostaining quantification, and densitometry data are shown as average ± standard deviation from three independent experiments. Statistical significance was assessed using Student’s *t* -test for planned comparisons or one-way ANOVA where indicated; *P* < 0.05 was considered significant. Representative immunostaining images and immunoblotting data are shown from three independent experiments.

## Data Availability

Description: Extended data 1. Additional controls and representative images for IRF9 immunostaining. (A) L929 cells were fixed, permeabilized, and processed for IRF9 immunostaining using a rabbit anti-IRF9 primary antibody (Abcam) followed by an Alexa Fluor 488-conjugated goat anti-rabbit IgG secondary antibody, or with secondary antibody alone after omission of the primary antibody. Nuclei were counterstained with DAPI. Minimal fluorescence signal was detected in the absence of primary antibody. (B) Additional representative images from independent experiments showing nuclear hyperaccumulation of IRF9 in T1L-infected L929 cells. (C) Immunofluorescence images of mock- and T1L-infected AD-293 cells showing nuclear accumulation of IRF9 in T1L-infected cells.. Resource Type: Dataset. DOI: https://doi.org/10.22002/1t8np-pfd65
